# Macrophages and Leydig Cells in Testicular Biopsies of Azoospermic Men

**DOI:** 10.1155/2014/828697

**Published:** 2014-05-04

**Authors:** Trpimir Goluža, Alexander Boscanin, Jessica Cvetko, Viviana Kozina, Marin Kosović, Maja Marija Bernat, Miro Kasum, Željko Kaštelan, Davor Ježek

**Affiliations:** ^1^Clinic for Gynaecology and Obstetrics, Clinical Hospital Centre Zagreb, School of Medicine, University of Zagreb, Petrova 13, 10000 Zagreb, Croatia; ^2^Loma Linda University, 11060 Anderson Street, Loma Linda, CA 92350, USA; ^3^Department of Histology and Embryology, School of Medicine, University of Zagreb, Šalata 3, 10000 Zagreb, Croatia; ^4^Department of Physics and Biophysics, School of Medicine, University of Zagreb, Šalata 3, 10000 Zagreb, Croatia; ^5^Clinic for Urology, Clinical Hospital Centre Zagreb, School of Medicine, University of Zagreb, Kišpatićeva 12, 10000 Zagreb, Croatia; ^6^Department for Transfusion Medicine and Transplantation Biology, Clinical Hospital Centre Zagreb, School of Medicine, University of Zagreb, Kišpatićeva 12, 10000 Zagreb, Croatia

## Abstract

A number of studies have indicated that testicular macrophages play an important role in regulating steroidogenesis of Leydig cells and maintain homeostasis within the testis. The current paper deals with macrophages (CD68 positive cells) and Leydig cells in patients with nonobstructive azoospermia (NOA). Methods employed included histological analysis on semi- and ultrathin sections, immunohistochemistry, morphometry, and hormone analysis in the blood serum. Histological analysis pointed out certain structural changes of macrophages and Leydig cells in NOA group of patients when compared to controls. In the testis interstitium, an increased presence of CD68 positive cells has been noted. Leydig cells in NOA patients displayed a kind of a mosaic picture across the same bioptic sample: both normal and damaged Leydig cells with pronounced vacuolisation and various intensity of expression of testosterone have been observed. Stereological analysis indicated a significant increase in volume density of both CD68 positive and vacuolated Leydig cells and a positive correlation between the volume densities of these cell types. The continuous gonadotropin overstimulation of Leydig cells, together with a negative paracrine action of macrophages, could result in the damage of steroidogenesis and deficit of testosterone *in situ*.

## 1. Introduction

Within the mammalian testis, there is a significant population of macrophages situated in the interstitial compartment. They have Fc and complement receptors, express macrophage-specific markers, produce interleukins, phagocytose and kill pathogenic organisms. These cells also incorporate trypan blue, plutonium, and demonstrate acid phosphatase and nonspecific esterase activity [1–7]. Morphological studies on the rat testis have revealed a specific ultrastructural coupling of macrophages and Leydig cells. Namely, Leydig cells project slender cytoplasmic processes towards deep coated channels of macrophages. The channels are limited by an electron dense part of the macrophage membrane. It can be supposed that these channels could be sites of an intensive exchange of molecules/signals between the two cell populations [1, 8, 9]. In the adult normal human testis, macrophages reside in the testis interstitium and are by no means encountered within seminiferous tubules [10]. However, in the biopsies of infertile men, these cells (apart from their interstitial location) can be seen within the lamina propria and seminiferous epithelium and/or lumen of the tubules [10–12]. Leydig cells are the major source of testosterone [13] but also are capable of producing many nonsteroidal factors like *β*-endorphin [14] and prodynorphin [15], which are known to act as paracrine factors affecting macrophage function.

Infertility affects in average 15–20% couples and is, therefore, a major health problem. Incidence of male infertility is rising due to various genetic, infectious, and environmental factors. The most difficult patients to treat are those presenting with azoospermia (no spermatozoa in the ejaculate). Approximately 8% of infertile men are diagnosed either with obstructive (OA) or nonobstructive azoospermia (NOA) and are frequently subjected to (micro)surgical spermatozoa retrieval (testicular biopsy) and testicular sperm extraction (TESE). In general, OA is characterized by an intact testicular parenchyma, maintained production of spermatozoa within seminiferous tubules, and normal morphology of the interstitial tissue. Yet, after a longer period of obstruction, some macrophages could be found within the seminiferous epithelium [16, 17]. NOA cases, however, demonstrate various degrees of seminiferous tubules damage: hypospermatogenesis, disturbance of spermatogenic cells maturation at the level of spermatids (spermatid “stop”), or spermatocytes (spermatocyte “stop”) up to drastic pathological changes such as Sertoli cells only syndrome and/or tubular fibrosis [18]. Sometimes, in the histological sections of NOA testicular biopsies, a combination of the above-mentioned pathology can be seen, recognized as a “mixed atrophy” of seminiferous tubules [19]. In the case of NOA, the structure of Leydig cells and the production of testosterone are frequently changed when compared to controls [20–22]. In the biopsies of infertile men, Leydig cells are often hypertrophic and/or hyperplastic and diffusely arranged around hypoplastic seminiferous tubules [23, 24]. The levels of testosterone could be either decreased or increased, depending on the nature of Leydig cells changes within the gonad. If Leydig cells are poorly differentiated from their mesenchymal precursors, they are not capable of maintaining the normal testosterone production. However, in many cases testosterone levels are maintained because Leydig cells are sufficiently differentiated and stimulated by LH. FSH and LH are frequently increased in infertile men with disturbance of spermatogenesis [25, 26]. In addition, the presence of mast cells in such bioptic material proved to be abundant [27]. These cells are found to be increased in various forms of NOA, including mixed atrophy [28–30]. Study of Bergh [31] demonstrated that macrophages and Leydig cells responded to unilateral cryptorchidism in a similar fashion. In the cryptorchid testis, both macrophages and Leydig cells were reduced in size and number.

Recent papers on testicular macrophages and Leydig cells interactions mostly deal with animal or* in vitro* models [32–35]. Since nonobstructive azoospermia is the form of male infertility largely difficult to treat, the aim of our study was to investigate morphological features of macrophages and Leydig cells in testicular biopsies of infertile men that were affected by that particular disorder. Moreover, we wanted to draw a parallel between the expression of testosterone production/expression* in situ* with morphological characteristics of macrophages and Leydig cells, as well as the levels of testosterone and gonadotropins in the blood serum of azoospermic patients.

## 2. Materials and Methods

### 2.1. Testicular Biopsy

Overall, 120 patients with azoospermia that consulted andrologist for male infertility in the period from 1998 to 2006 at the Clinic of Urology (University of Zagreb, School of Medicine, University Centre Zagreb) were included into the study. All patients were subjected to an open biopsy of the testis [36, 37]. Prior to the biopsy, patients gave their written and informed consent to surgery and biopsy examination. The current study has been approved by an appropriate ethical committee. A detailed diagnostic procedure preceded the testicular biopsy, including minimum 2-3 semen analyses in order to confirm azoospermia. The testis volume has been determined by ultrasound. Whenever possible, a bilateral biopsy was performed. Briefly, an incision of 8–10 mm in length in the t. albuginea of the testis has been made. This incision allowed 4-5 testicular lobules to be included into the biopsy. The protruding testicular tissue was dissected using surgical microscissors. Typically, 5 pieces were taken from different parts of the male gonad. After dissection, 4 pieces were immediately fixed in Bouin's fluid and 1 piece in a buffered 5.5% glutaraldehyde [18]. Careful histological analysis identified twelve patients with fully preserved spermatogenesis according to the Johnsen's score [25] and normal morphology of the interstitial tissue. Based on their clinical presentation and histology analysis, those patients were diagnosed with obstructive azoospermia (OA) and formed a control group (*N* = 12). The average age of patients was 34 years (range 24–37 years). The rest of the patients (*N* = 108) displayed various degrees of damage of a testicular parenchyma: hypospermatogenesis, spermatid and spermatocyte “stop,” spermatogonia only syndrome, picture of Sertoli cells only syndrome, tubular sclerosis, or combination of the aforementioned testicular disorders known as “mixed atrophy” [19]. Those patients were diagnosed with nonobstructive azoospermia (NOA). The average age of patients was 32 years (range 22–43 years).

### 2.2. Tissue Processing

Tissue fixed in glutaraldehyde was rinsed several times in 0.05 M phosphate buffer (pH = 7.1–7.4, 800 mOsm) and postfixed with 1% OS0_4_. The tissue was then dehydrated in a series of ascending alcohol concentrations. After a routine histological procedure, the testicular tissue was embedded in Durcupan (Agar). Semithin sections (section thickness = 0.9 *μ*m) were made by Reichert ultramicrotome and stained with 1% toluidine blue. Sections were covered with a drop of Merkoglass (Merck) and a coverslip. Ultrathin sections were made (Reichert ultramicrotome, section thickness = 70 nm), contrasted with lead citrate and uranyl acetate, and examined by a transmission electron microscope Zeiss 902A (Centre for Electron Microscopy, Medical School University of Zagreb).

Small pieces of testicular tissue (similar to the size of a rice) fixed in Bouin's fluid were dehydrated. For a morphometric (stereological) part of the study, the tissue was allowed to be oriented in a random fashion and embedded in paraffin blocks. Each block was trimmed and oriented (also in a random fashion) for cutting. The blocks were then cut extensively by a rotary microtome (Leitz). Part of serial sections for a routine bright field microscopy (section thickness = 4 *μ*m) was placed on slides and stained with hemalaun and eosin (H&E). After staining, slides were inspected by a binocular microscope Eclipse 200 (Nikon). The unstained sections were prepared for immunohistochemistry.

### 2.3. Immunohistochemistry

Unstained paraffin sections were placed on silane-coated slides. Slides were then incubated for 20 minutes at 60°C. Sections were deparaffinized and incubated for 3 × 5 minutes in 10 mmol/L citrate buffer (pH 6.0) in a microwave oven at 800 W and 400 W powers for antigen retrieval [38]. Subsequently, tissue slides were washed with PBS buffer (pH 7.2) and the endogenous peroxidase activity was blocked by a 5-minute treatment with H_2_O_2_. Slides were then washed with PBS buffer and preadsorbed for 15 min with 20% mouse serum. Afterwards, the slides were incubated in a humid chamber for 30 minutes with a primary antibody at a room temperature. Following primary antibodies were used according to the manufacturer's instructions: CD68 (macrophage/monocyte marker, 1 : 100, Dako, Glostrup, Denmark) and testosterone (1 : 50, Biogenex, San Ramon, USA). After washing in PBS buffer, the secondary biotinylated antibody (or the appropriate kit with an envision complex) was added for 30-minute incubation. Slides were then washed with PBS buffer and treated with streptavidin-horseradish peroxidase for 30 minutes. Tissue sections were washed once more in PBS buffer and then chromogen 3,3′-diaminobenzidine was added for 5 minutes. Slides were washed in distilled water, stained with hemalaun for 1 minute, washed with water, dehydrated with alcohol (96%), cleared with xylene, and mounted with Entellan (Merck, Darmstadt, Germany). As a negative control, omission of primary antibodies was used.

### 2.4. Morphometric (Stereological Analysis)

For a stereological analysis, a nonbiased Weibel's 42-point multipurpose test system was applied at a magnification of ×400 [39]. The length of the test lines was 0.045 mm, and the test surface area was 0.0736 mm^2^ for each analyzed microscopic field. The slides that underwent immunohistochemistry staining were placed under the microscope. They were focused (without refocusing). In order to determine the volume (or volume density, *V*_*v*_) of immunopositive cells (CD68 positive cells and testosterone producing cells) in a unit volume of testicular tissue, a point counting method has been used. To determine *V*_*v*_ the following formula was applied [40–42]:

(1) $$\begin{array}{c} V_{v}=\frac{P_{c}}{P_{t}}, \end{array}$$

where *P*_*c*_ is number of hits on positive cells whereas *P*_*t*_ is a number of test points (*P*_*t*_ = 42). A pilot stereological measurement of *V*_*v*_ has been made in order to determine the number of microscopic fields (*n*) needed for a statistically reliable data assessment. The pilot measurement has been carried out on 20 microscopic fields. After this preliminary measurement, de Hoff's formula was applied [40–42]:

(2) $$\begin{array}{c} n={\left(20\times \frac{s}{x}\right)}^{2}, \end{array}$$

where *x* is the mean value of *V*_*v*_ and *s* is the standard deviation. In our case, the number of microscopic fields to be assessed for each patient was 20–50, depending on the results of the pilot measurement/s.

### 2.5. FSH, LH, and Testosterone Blood Serum Levels

The levels of follicle stimulating hormone (FSH), luteinizing hormone (LH), and testosterone in blood serum were determined using standard reagents (kits) provided by Ortho-Clinical Diagnostics (Johnson & Johnson, Amersham, UK). A Vitros ECi device has been used. Reagents contained a set of 100 microwells with antibody for the appropriate hormone/s bound to horseradish peroxidase, antibody to hormone/s bound to biotin, calibrators, signal reagent, and washing solution. An enhanced chemiluminescence method has been applied.

### 2.6. Statistical Analysis

Data were statistically analysed using Statistica 10.0 software developed by StatSoft. The statistically significant difference between the control and NOA group of patients was determined using nonpaired Student's* t*-test. *P* < 0.05 indicated a statistically significant difference between the two groups. Based on the aforementioned test and the results of general statistics package, graphs and a table representing mean values and standard errors of means were generated. In addition, Pearson's correlation coefficient between volume density of CD68 positive cells (*V*_*v*_ CD68+) and vacuolated Leydig cells (*V*_*v*_ Lc) was determined along with statistical significance of correlation.

## 3. Results

### 3.1. Qualitative Histological Analysis on Semithin Sections

**Figure 1:**
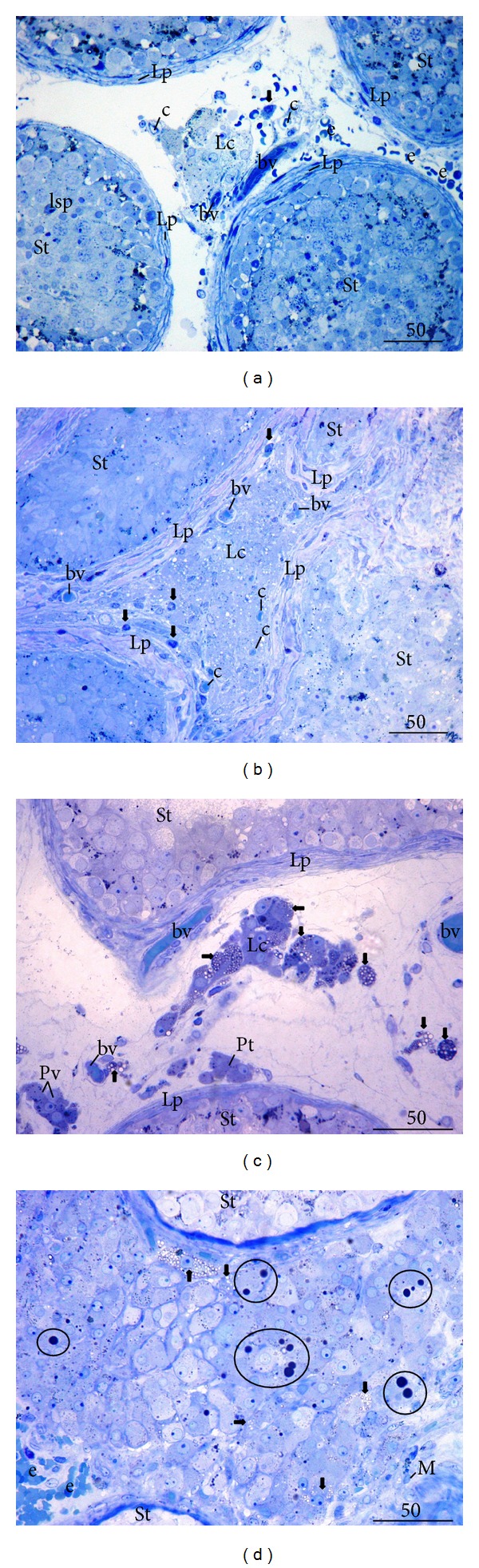
Semithin sections of the control testicular biopsy (obstructive azoospermia case, OA) (a) and biopsies from patients with nonobstructive azoospermia (NOA) ((b)–(d)). (a) Between several seminiferous tubules (St) with regular spermatogenesis, a part of the testicular interstitium with a cluster of Leydig cells (Lc) is visible. Leydig cells have a regular, round nucleus surrounded by an abundant cytoplasm. In the close proximity of these cells, a presumptive macrophage (**↓**) and several small blood vessels (bv) including capillaries (c) are noted (Lp, lamina propria; lsp, late spermatid; e, extravasated erythrocytes). (b) A part of testicular biopsy from a patient with NOA. Seminiferous tubules (St) are lined with Sertoli cells only and have a thickened lamina propria (Lp). Leydig cells (Lc) in the middle demonstrate a high vacuolisation of the cytoplasm. Presumptive macrophages (**↓**) are arranged at the periphery of seminiferous tubules or between Leydig cells. Several small blood vessels (bv) as well as capillaries (c) are also found. (c) Testicular biopsy from a patient with a spermatocyte “stop.” According to their location/related to seminiferous tubules (St) and blood vessels (bv)/, Leydig cells can be subdivided into peritubular (Pt) and perivascular (Pv). These cells demonstrate regular morphology, in contrast to many vacuolated Leydig cells (**→**) in the center (Lc) (Lp, lamina propria). (d) Testicular biopsy (NOA patient, spermatocyte “stop”) with Leydig cell hypertrophy/hyperplasia. Within the abundant population of Leydig cells, those with vacuolated cytoplasm (**→**) and large lipid droplets (encircled areas) can be observed (St, seminiferous tubules; M, presumptive macrophage; e, extravasated erythrocytes) (Toluidine blue, ×400/(a), (b)/, ×600/(c), (d)/, scale bar = 50 *μ*m).

Control biopsies from patients with OA displayed regular morphology of the testicular parenchyma: seminiferous tubules had regular diameter (200–220 *μ*m), normal thickness of the lamina propria (10–15 *μ*m), and basement membrane and well-developed seminiferous epithelium. The epithelium consisted of supportive Sertoli cells and all kinds of spermatogenic cells: spermatogonia, spermatocytes, round and elongated spermatids, and spermatozoa (Figure 1(a)). The loose connective tissue of the interstitial compartment predominantly consisted of fibrocytes and fibroblasts. Within this tissue, clusters of Leydig cells were noted. Leydig cells had conspicuous round or oval nucleus with sometimes prominent nucleolus. Their cytoplasm was abundant, bearing few lipid droplets and sometimes one or two vacuoles. Small blood vessels like arterioles, venules, and capillaries were found interwoven in the Leydig cell clusters, in the close proximity of seminiferous tubules, or in the wall of the lamina propria (which was especially true for capillaries) (Figure 1(a)). Depending on their location, Leydig cells could be subdivided into peritubular (closer to seminiferous tubules) or perivascular (closer to blood vessels in the middle of the interstitial space). Large cells residing within the interstitium, close to Leydig cells, with a kidney-shaped or indented nucleus and well-developed cytoplasm were classified as “presumptive macrophages.” Frequently, within the interstitial space, extravasated erythrocytes were observed as a consequence of a surgical procedure (Figure 1(a)).

NOA group of patients was represented by various histological pictures: within some biopsies, small number of mature or elongated spermatids together with abundant presence of round spermatids and other spermatogenic cells were seen (spermatid “stop”); some biopsies were depleted of spermatids and spermatogenesis went up to the spermatocyte stage (spermatocyte “stop”). In some cases, only spermatogonia and Sertoli cells were found within the seminiferous epithelium (spermatogonia only) or the epithelium consisted only of Sertoli cells (“Sertoli cells only” syndrome). Small proportion of NOA patients (3%) had tubular sclerosis where all seminiferous tubules were transformed into the fibrous tissue or “tubular shadows.” However, in the vast majority of the cases (68% of NOA patients) a combination of the above-mentioned histological pictures could be recorded, which is known as a “mixed atrophy” of seminiferous tubules.

NOA patients also displayed a variety of histological changes within the interstitial tissue (which accompanied various degrees of the damage of seminiferous tubules). Frequently, the loose connective tissue of the interstitial compartment was densely packed with fibrocytes, macrophages, and Leydig cells together with small blood vessels (Figure 1(b)). In general, NOA patients had a kind of mosaic Leydig cells picture across the same bioptic sample: in some areas Leydig cells had a regular appearance; in other areas, their morphology has been changed (Figures 1(b)–1(d)). The interstitial tissue sometimes bore smaller Leydig cells in size (when compared to the control group). Within the cytoplasm of these cells, numerous small translucent vacuoles were identified (Figure 1(b)). In other areas (i.e., of the same biopsy) Leydig cells were of mixed morphology: some preserved their typical round or oval nucleus with rich cytoplasm and few small lipid droplets. Others, however, had plentiful vacuoles and small or large lipid droplets (Figures 1(b)–1(d)). Sometimes, a focal or diffuse Leydig cells hypertrophy was observed. Seminiferous tubules were surrounded by many large Leydig cells. In the cytoplasm of these hypertrophic cells, numerous vacuoles and/or large lipid droplets were identified. The vacuoles were either located in one part of or diffusely spread throughout the cell cytoplasm (Figure 1(d)). In semithin sections, presumptive macrophages were found in the vicinity of the lamina propria of seminiferous tubules (or within it) or intermingled with Leydig cells (Figure 1(b)). Sometimes, their cytoplasm was loaded with phagocytized material (Figure 1(d)).

### 3.2. Transmission Electron Microscopy (TEM)

**Figure 2:**
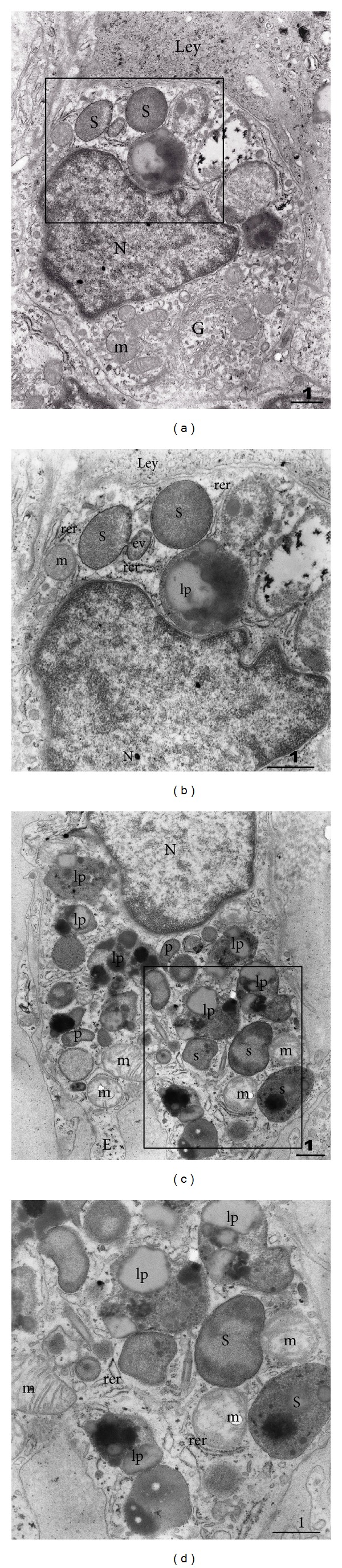
Transmission electron microscopy (TEM) of testicular macrophages in the control biopsy ((a), (b)) and in the case of NOA ((c) and (d)). (a) Macrophage with indented nucleus (N) is facing the neighbouring Leydig cell (Ley). Perinuclear region bears a well-developed Golgi apparatus (G). Several mitochondria (m) are found in the vicinity of the apparatus. Within another part of the cytoplasm, secondary lysosomes (s) are visible. The framed area is shown in (b). (b) The cytoplasm of the macrophage opposing Leydig cell (Ley) is rich in secondary lysosomes (s) and lipofuscin bodies (lp). Endocytic vacuole (ev) is in a process of a fusion with one secondary lysosome (N, nucleus; m, mitochondrion; rer, rough endoplasmic reticulum). (c) A macrophage from NOA case. A part of the nucleus (N) is visible, surrounded by an organelle-rich cytoplasm that bears a lot of primary (p) and secondary (s) lysosomes. Numerous lipofuscin bodies and phagosomes (lp) demonstrate intensive phagocytic activity of the cell (m, mitochondria; E, cellular extension; framed area represented in (d)). (d) Between secondary lysosomes (s) and lipofuscin bodies (lp) some scattered cisternae of rough endoplasmic reticulum (rer) and mitochondria (m) could be noted (TEM, original magnification ×7.000/(a), (c)/, ×12.000/(b), (d)/; scale bar = 1 *μ*m).

In control biopsies, testicular macrophages (that were occasionally present within clusters of Leydig cells or in their proximity) had an indented nucleus with plenty of euchromatin. Few patches of heterochromatin were found within the euchromatin or typically associated with the nuclear membrane. A prominent Golgi apparatus, a lot of mitochondria with cristae, cisternae of predominantly rough endoplasmic reticulum, and few lipid droplets were identified within the cytoplasm. In addition, macrophages in control biopsies had moderate number of primary and secondary lysosomes and occasional residual/lipofuscin bodies. The borders between macrophages and nearby Leydig cells were clearly defined by their cell membranes. No cellular extensions of Leydig cells towards macrophages were recorded (Figures 2(a) and 2(b)).

When compared to macrophages in control biopsies, the same type of cell in NOA group bore much more secondary lysosomes, residual and lipofuscin bodies, and cellular extensions (Figure 2(c)). Within secondary lysosomes and lipofuscin bodies, a variety of phagocytized material has been observed (Figure 2(d)).

**Figure 3:**
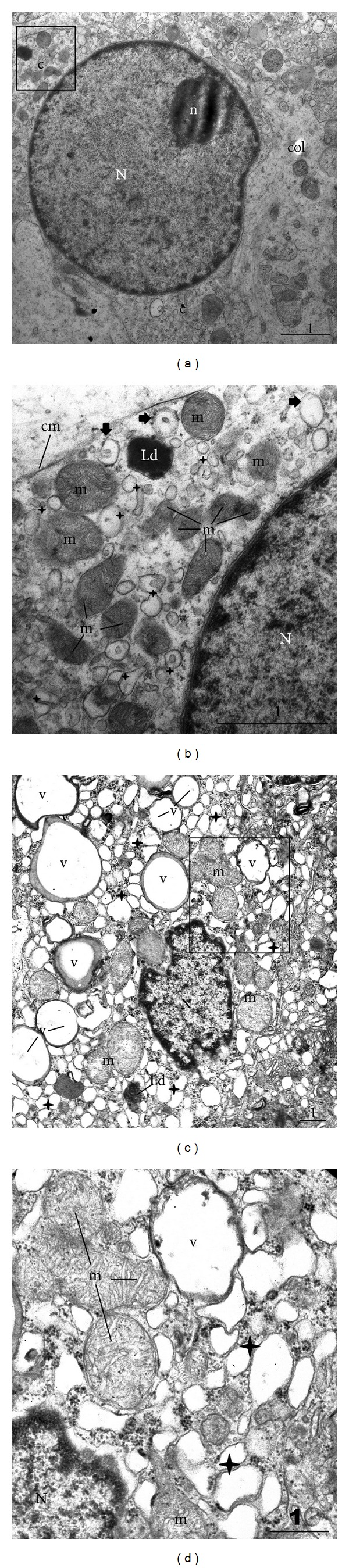
Transmission electron microscopy of a Leydig cell in the control biopsy ((a), (b)) and in the case of NOA ((c) and (d)). (a) Leydig cell from the control biopsy. Within a regular, rounded nucleus (N) a prominent nucleolus (n) is found. Cytoplasm (c) contains various organelles. The cell is surrounded by an extracellular matrix with collagen fibres (col). Framed area is represented in (b). (b) Part of the Leydig cell depicted in the previous picture. In the close proximity of the nucleus (N), numerous mitochondria (m) with tubular cristae, many cisternae of smooth endoplasmic reticulum (*✦*), and a lipid droplet (Ld) are visible. Occasional polyribosomes (**→**) are bound to few cisternae of rough endoplasmic reticulum (cm, cell membrane). (c) Leydig cell from the biopsy of NOA patient. The nucleus (N) is heavily indented and irregular. The cytoplasm bears a lot of vacuoles (v) with a low electron density material. Other typical organelles for a steroid-producing cell are also present: mitochondria (m), cisternae of smooth endoplasmic reticulum (*✦*), and sporadic lipid droplets (Ld). The framed area is represented in (d). (d) A part of Leydig cell shown in the previous figure, higher magnification. In the vicinity of the nucleus (N), a cell cytoplasm has a vacuole (v) with a kind of a unit membrane. The majority of the vacuole is translucent. However, the rim contains a thin area of the material of moderate electron density (also present in other vacuoles illustrated in (c)). Some of the cisternae of smooth endoplasmic reticulum (*✦*) are wider than normal (m, mitochondria) (TEM, original magnification ×7.000/(a), (c)/, ×20.000/(b), (d)/; scale bar = 1 *μ*m).

Leydig cells of the control group were recognized by a large, round nucleus and frequently prominent nucleolus. The nucleus was regular (mostly without indentations), rich with euchromatin whereas heterochromatin was arranged along the nuclear membrane. Well-developed cytoplasm contained a lot of organelles. Cells were surrounded by an extracellular matrix rich in collagen fibres (Figure 3(a)). Inside the cytoplasm, a lot of cisternae of smooth endoplasmic reticulum were seen. Small numbers of cisternae of rough endoplasmic reticulum were also observed. Leydig cells were rich in mitochondria with tubular cristae. Few lipid droplets and one or two vacuoles were recorded as well (Figure 3(b)).

The “mosaic picture” of Leydig cells in NOA biopsies was also confirmed by TEM. Significant number of cells was normal in appearance, with typical ultrastructure as the cells in control biopsies. In contrast to those cells, some Leydig cells in the group of patients with NOA had a nucleus with pronounced indentations. The euchromatin had more patches of heterochromatin and nucleolus was frequently absent (Figure 3(c)). Furthermore, changed cells had much more lipid droplets varying in size and a lot of vacuoles. Vacuoles seemed to have a unit membrane and content with a low electron density, apart from the thin area along the rim of the vacuole. That area frequently displayed material of a moderate electron density. The majority of cisternae of smooth endoplasmic reticulum were wider than normal, although the same cell could have areas of the cytoplasm with normal cisternae as well. The morphology of mitochondria was found to be preserved (Figures 3(c) and 3(d)).

### 3.3. Results of Immunohistochemistry (IHC) Analysis

**Figure 4:**
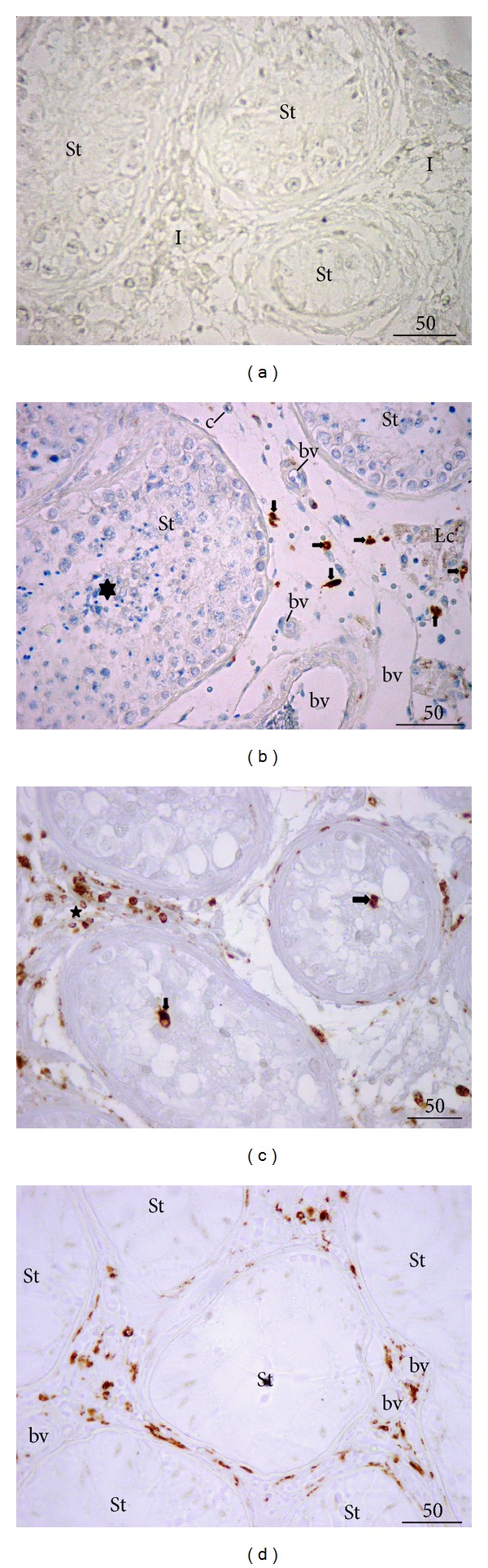
Expression of a monocyte/macrophage marker, CD68. (a) Control of immunohistochemistry (IHC) reaction, preadsorption, and omission of a primary antibody. No signal is present either in the testicular interstitium (I) or seminiferous tubules (St). (b) Control biopsy with full spermatogenesis. Within the seminiferous tubules (St), a lot of elongated spermatids (★) are visible. Between seminiferous tubules, a moderate presence of macrophages (CD68 positive cells,** →**) is notable. None of these cells are found within seminiferous tubules (Lc, cluster of Leydig cells; bv, blood vessels). (c) A biopsy from a patient with NOA. Seminiferous tubules (lined with Sertoli cells only or occasional spermatogonia) are surrounded by a loose connective tissue heavily loaded with CD68 positive cells (★). Some of these cells are found attached to the lamina propria, or invading the lumen of the tubules (**→**). (d) NOA patient. Seminiferous tubules (St) show a picture of Sertoli cells only syndrome. The testis interstitium is rich with CD68 positive cells (3,3′-diaminobenzidine (DAB), hemalaun counterstain, ×400, scale bar = 50 *μ*m).

Control slides with preadsorption and omission of primary antibodies (either for CD68 or testosterone) proved to be negative. No positive cells were found in seminiferous tubules and testicular interstitial compartment (Figure 4(a)). Within control testicular biopsies, CD68 was expressed in a moderate number of cells within the loose connective tissue of the interstitium. CD68 positive cells were surrounded by other connective tissue cells. Few of such cells were found in the vicinity of the lamina propria of seminiferous tubules and clusters of Leydig cells (Figure 4(b)). No CD68 positive cell was found within the seminiferous tubules whatsoever (Figure 4(b)).

In the biopsies of NOA patients, CD68 positive cells were abundant in the interstitial tissue. They infiltrated areas with Leydig cells as well as the lamina propria of some seminiferous tubules. Moreover, these cells were found crossing the tubular wall and invading the seminiferous epithelium as well as the tubular lumen. CD68 positive cells in the proximity of blood vessels were also recorded (Figures 4(c) and 4(d)).

**Figure 5:**
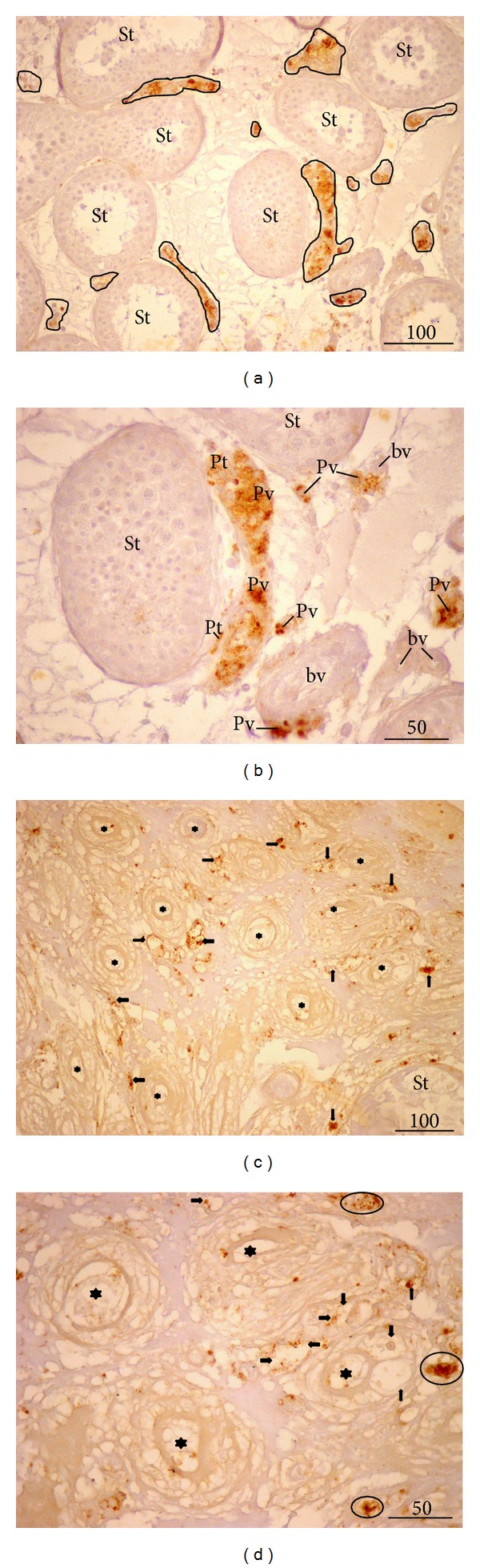
Testosterone expression* in situ*. (a) Control biopsy. Larger or smaller groups of Leydig cells are positive to testosterone (encircled areas) (St, seminiferous tubules). (b) Testosterone expression, details of the previous figure. A strong signal is present both in peritubular (Pt) as well as perivascular Leydig cells (Pv) (St, seminiferous tubules; bv, blood vessels). (c) NOA, testicular biopsy with tubular sclerosis (★) and intensive vacuolisation of Leydig cells. An irregular expression of testosterone (**→**) is notable (St, seminiferous tubule). (d) NOA, higher magnification. Some Leydig cells demonstrate highly scattered and irregular positive signal (**→**); in contrast, few of them possess normal or very strong signal (encircled cells) (★, fibrosed tubules, the so-called “tubular shadows”) (3,3′-diaminobenzidine (DAB), hemalaun counterstain, ×200, scale bar = 100 *μ*m/(a), (c)/; ×400, scale bar = 50 *μ*m/(b), (d)/).

In control biopsies, testosterone positive cells were arranged either in larger or smaller groups in the interstitial compartment (Figure 5(a)). Both peritubular and perivascular Leydig cells were found to be positive for testosterone. Although varying in intensity, the signal was homogenously distributed throughout the whole cluster/s of cells (Figures 5(a) and 5(b)). Leydig cells of NOA biopsies displayed a kind of mixed expression of testosterone. Some of the cells had normal intensity of the signal, whereas some demonstrated a weak and irregular expression of testosterone (Figures 5(c) and 5(d)). Moreover, in some cells the signal was rather strong with a kind of an overexpression of testosterone (Figure 5(d)). The irregular and inhomogeneous signal was especially present in samples where (in semithin sections) many Leydig cells had vacuoles in their cytoplasm.

### 3.4. Results of Quantitative Analysis: Testis Volume, Status of Spermatogenesis, Stereology Data, and Hormones Levels

Testis volume in patients with nonobstructive azoospermia was significantly decreased in comparison to the control group (*P* < 0.001) (Table 1). The same goes for the status of spermatogenesis measured by Johnsen's score. In NOA group of patients, the spermatogenesis was significantly impaired when compared to controls (*P* < 0.001) (Table 1).

**Figure 6:**
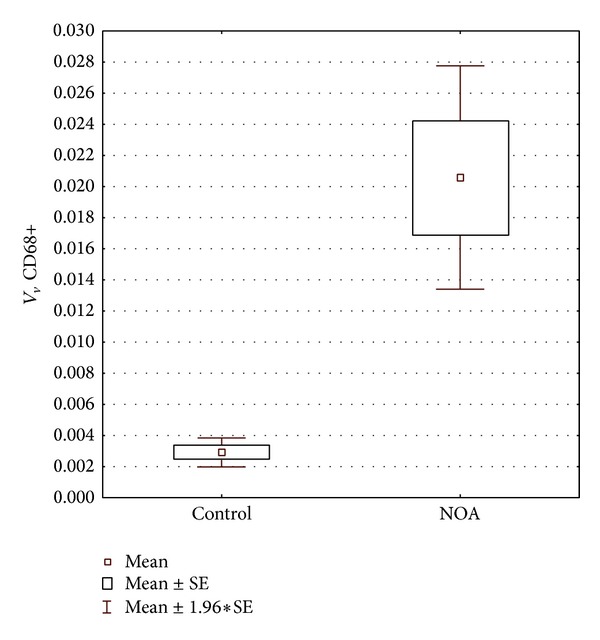
Volume density of CD68 positive cells (*V*_*v*_ CD68+) in control and NOA group of patients. Volume density of these cells in NOA group is significantly increased (*P* < 0.001).

**Figure 7:**
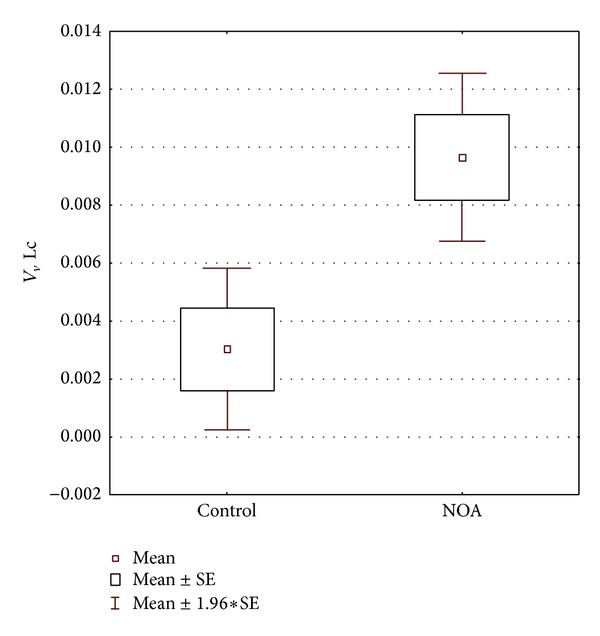
Volume density of vacuolated Leydig cells in control and NOA group. Volume is significantly increased in the group of patients with nonobstructive azoospermia (*P* < 0.05).

**Figure 8:**
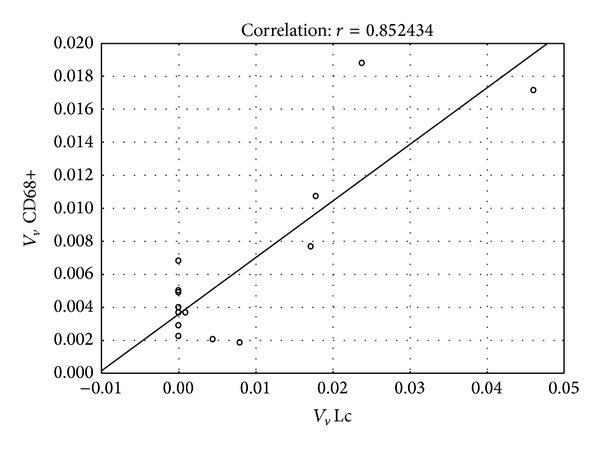
Correlation between volume density of CD68 positive cells and volume density of vacuolated Leydig cells. A statistically significant positive correlation has been found (*r* = 0.852434, *P* < 0.001).

**Figure 9:**
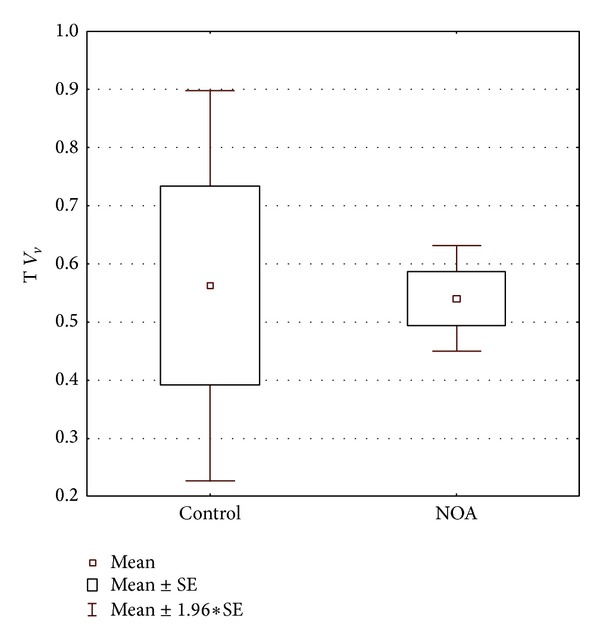
Volume density of testosterone immunopositive cells in control and NOA group. There is no statistical difference between the investigated groups (*P* > 0.05).

Volume density determined for CD68 positive cells showed statistically significant difference between the control and NOA group of patients. In NOA group, this stereological variable had significantly higher value than in controls (*P* < 0.001) (Figure 6). NOA group also displayed a significantly higher volume of vacuolated Leydig cells when compared to the control group (*P* < 0.05) (Figure 7). The correlation analysis pointed out a significant positive correlation between the volume density of CD68 positive cells and the volume density of Leydig cells with vacuoles (*r* = 0.852434, *P* < 0.001) (Figure 8). In contrast to the results described above, stereological analysis showed no statistically significant difference in the volume density of cells positive to testosterone between control and NOA group of patients (*P* > 0.05), although the volume density of cells expressing testosterone* in situ* was higher in the control group of biopsies (Figure 9).

The analysis of blood serum levels of gonadotropins (FSH, LH) demonstrated significantly higher levels of these hormones in NOA group of patients (*P* < 0.01, *P* < 0.001, resp.). Control group of patients had normal levels of gonadotropins (Table 1). Testosterone serum levels were within the normal range in the control group of patients. The vast majority of NOA patients had normal levels of testosterone as well. However, in a number of NOA patients (*N* = 11), testosterone levels lower than normal range have been noted. In contrast, small group of patients (*N* = 3) had increased levels of testosterone. There was no statistically significant difference between control and NOA group in testosterone levels (*P* > 0.05) (Table 1).

## 4. Discussion

There is a growing body of evidence that testicular macrophages play an important role in regulating steroidogenesis of Leydig cells and maintain homeostasis within the testis [43–45]. Under normal physiological and noninflammatory conditions, macrophages play an important role in Leydig cell development. If macrophages are absent from the testicular interstitium, Leydig cells do not succeed in developing normally, which suggests that macrophages provide important growth and differentiation factors for Leydig cells. In infertile men with damage of spermatogenesis and/or chronic orchitis, when macrophages are activated and secrete an array of inflammatory mediators, Leydig cell steroidogenesis is inhibited [43].

Despite a significant progress in the area of reproductive medicine and high curing rates, NOA remains to be a considerable problem. As pointed by our results as well, NOA is often characterized by marked pathological changes in both tubular and interstitial compartment. NOA patients could suffer from a testosterone deficit. If the levels of testosterone drop below 15 nmol/L, a change in mood, depression, and a loss of libido have been noted. The levels of testosterone below 8 nmol/L cause erectile dysfunction. Other symptoms include adiposity, insomnia, concentration deficit, diabetes mellitus type 2, and, in the long run, osteoporosis [46, 47]. Thus, it is important to have an insight into the macrophage-Leydig cells interaction in NOA patients. The current study aims to provide data on morphological changes of Leydig cells (and decreased production of testosterone* in situ*) in the presence of increased macrophage population (CD68 positive cells) in NOA patients, which could also clarify the above-mentioned clinical observations.

In our study, the vast majority of NOA patients had a significantly decreased testis volume. This was in a way supported by data assessed on semithin sections, where a pronounced loss of various spermatogenic cells has been recorded. When the status of spermatogenesis has been assessed by Johnsen's score, this score was significantly lower in NOA patients. Semithin sections have also provided an insight into the morphology of interstitial tissue (that is frequently neglected in the routine work because of the search for spermatozoa in seminiferous tubules). In semithin sections of testicular biopsies of NOA patients, a pronounced vacuolisation of some Leydig cells and a notable presence of macrophages loaded with phagocytized material have been recorded. Macrophages were present not only in the close proximity of seminiferous tubules, but they were also increasingly “intermingled” with Leydig cells. The above-mentioned observations were strongly supported by our immunohistochemistry and morphometric data. Thus, in NOA group, multitudinous CD68 positive cells were found in the interstitial compartment, infiltrating the lamina propria of seminiferous tubules as well as seminiferous epithelium. Since CD68 antibody identifies monocyte/macrophage lineage, the observed CD68 positive cells are very likely recruited from blood and infiltrated interstitial and tubular testis compartments, in addition to resident macrophages that were already present in the loose connective tissue of the testis.

Namely, it seems that the population of testicular macrophages, at least in the rat, is not a homogenous one. The majority of testicular macrophages exhibit a cell-surface antigen, which is recognised by monoclonal antibody ED2. This antigen is expressed exclusively on fixed tissue-resident macrophages [48, 49]. However, a small population of testicular macrophages do not have detectable ED2 antigen. These macrophages can be identified because they are positive for the antigen recognised by antibody ED1 (CD68), a marker of both circulating monocytes and macrophages. Approximately half of the ED2 positive resident testicular macrophages lack detectable expression of ED1. It is very likely that this marker is lost after the cells become resident in the testis [48, 49]. Freshly isolated testicular macrophages from normal rats produce relatively low levels of the inflammatory cytokines interleukin-1 beta and tumour necrosis factor-*α* in response to various stimuli [50–52]. These data suggest that testicular macrophages, at least in the rat, are poorly proinflammatory. In humans, interleukin-1 alpha has been related with orchitis, relapse of acute lymphoblastic leukemia in the testis, and infertility disorders in men [53].

The results of our study, in particular its immunohistochemistry and morphometry part, concur to those presented by Frungieri et al. [11], where an increased number of CD68 positive cells were clearly identified in the loose connective tissue of the interstitium and in the lamina propria of hypoplastic seminiferous tubules as well as within the seminiferous epithelium and tubular lumen. Based on these findings, it has been postulated that testicular macrophages both directly (via phagocytosis) or indirectly (via paracrine modulators) have been involved in the regulation of steroidogenesis. Our study clearly pointed out some changes in the Leydig cell fine structure in the increased presence of CD68 positive cells. Stereological analysis emphasized that there was an increased presence of CD68 positive cells and vacuolated Leydig cells in NOA group of patients. Moreover, a positive correlation between these two cell groups has been found: the more the CD68 positive cells, the more the vacuolated Leydig cells.

Our results, based on the histological analysis of NOA Leydig cells in semithin sections, pointed out that these cells underwent both hypertrophy and hyperplasia. Apart from Leydig cells with large, abundant cytoplasm loaded with lipid droplets and/or vacuoles, there were also some smaller forms. These cells were elongated, much resembled to fibroblasts but expressed testosterone and, ultrastructurally, had cell organelles of steroid-producing cells. Frequently, across the same NOA biopsy, both such “types” of Leydig cells could be found. In the morphometric study of Tash et al. [24], it has been claimed that Leydig cells in NOA patients undergo hypertrophy only and their number remains constant when compared to OA cases. The measurements have been based on morphometric data and decreased testis volume of NOA patients, which was also presented in our study. However, the decrease of the testis volume is mainly due to spermatogenic cell loss and tubular hypoplasia, and, to the lesser extent, due to changes within the testis interstitium. The small “type” of Leydig cells that we noticed in semithin sections could be developed from mesenchymal precursors that are present in the loose connective tissue of the interstitial compartment. Thus, the question of Leydig cell hypertrophy/hyperplasia should be further explored.

A number of studies also pointed out the association between macrophages and mast cells and possible involvement of both cell types in the fibrotic changes of seminiferous tubules [11, 28–30]. In one of our previous papers [27] we presented similar observations, particularly in relation to NOA cases with “mixed atrophy” of seminiferous tubules. Although there are data on the interactions between macrophages and mast cells [11], the exact influence of mast cells on Leydig cells and steroidogenesis remains to be further investigated.

Our transmission electron microscopy analysis has demonstrated that macrophages of NOA patients contain a significant number of secondary lysosomes, residual and lipofuscin bodies, and cellular extensions. The ultrastructural analysis demonstrated the existence of large vacuoles within the cytoplasm of Leydig cells. The vacuoles were translucent with low electron density. They seemed to have a unit membrane and were accompanied by wide cisternae of smooth endoplasmic reticulum. The nature of vacuoles and their exact content remain to be further investigated. The vacuole could be a product of a fusion of several wide cisternae of smooth endoplasmic reticulum. At the rim of the vacuoles, along the unit membrane, a material of a moderate electron density has been frequently observed. It could be speculated that the above described vacuoles contain lipids or inhomogeneous material with lipids component/s. It should be mentioned at this point that, at the ultrastructural level, typical lipid droplets do not have a unit membrane. Moreover, vacuoles have been recorded either in one part of Leydig cell or they were diffusely arranged throughout the entire cytoplasm. Thus, normal cell machinery (especially smooth endoplasmic reticulum and mitochondria where important steps of steroidogenesis take place) is replaced by huge vacuoles. It can be speculated that the highly vacuolated Leydig cells with wide cisternae of smooth endoplasmic reticulum are only partially effective in terms of testosterone production. A negative influence of nearby located activated macrophages could add to testosterone deficiency* in situ*. This has been supported by our immunohistochemistry data (expression of testosterone). In NOA patients, some Leydig cells had a normal pattern of testosterone expression, similar to those in controls. In other cells (observed as highly vacuolated in semithin sections) the signal was largely irregular and decreased. One can speculate that vacuoles, which occupy a significant part of the cytoplasm, are negative for testosterone and cause the above-mentioned irregular pattern of expression or no expression at all. On the other hand, in some Leydig cells an overexpression of testosterone has been found.

The analysis of hormone levels in blood serum indicated high levels of gonadotropins (FSH, LH) in NOA patients. This concurs to the results of our previous study [25] and others dealing with NOA [54–56]. Thus, Leydig cells are continuously stimulated by LH. One can assume that the constant “bombardment” with gonadotropins, together with paracrine factors of macrophages, causes damage of Leydig cells. Overstimulation of these cells could provoke building of wide cisternae of smooth endoplasmic reticulum and huge vacuoles described in the current paper. All these events can create a kind of “burn-out” syndrome for Leydig cells. The deficit of testosterone* in situ* could act negatively on spermatogenesis, causing loss of spermatogenic cells and infertility. The results of our immunohistochemistry and morphometric analysis as well as testosterone levels in the blood support such a scenario. The measurement of the volume density of testosterone producing cells indicated no significant difference between control and NOA group, although the control group had slightly higher values of immunopositive Leydig cells. This lack of significant difference could be due to the (hyper)stimulation of LH. Thus, NOA patients have, in the vast majority of cases, normal values of testosterone. Overstimulated Leydig cells as well as those cells that preserved their normal structure and function (seen in semithin sections) could produce enough testosterone to maintain the normal serum levels. In addition, overstimulated Leydig cells could produce an excess of testosterone, which explains a small proportion of NOA patients that had higher levels of testosterone and overexpression of this hormone recorded in the immunohistochemistry part of our study. Finally, few NOA patients would have “exhausted,” burn-out Leydig cells and, therefore, lower levels of testosterone in blood than normal. Thus, in NOA patients, CD68 positive cells act negatively of Leydig cells. What is the exact trigger of such a pattern in NOA remains to be elucidated.

## 5. Conclusion

The current paper deals with macrophages (CD68 positive cells) and Leydig cells in patients with nonobstructive azoospermia (NOA). Histological analysis pointed out certain structural changes of macrophages and Leydig cells in NOA group of patients when compared to controls. These changes included an increased presence of CD68 positive cells. Leydig cells in NOA patients displayed a kind of a mosaic picture: both normal and damaged Leydig cells with pronounced vacuolisation and various intensity of expression of testosterone have been observed across the same bioptic samples. There was a positive correlation between the volume density of CD68 positive and vacuolated Leydig cells. It has been speculated that the continuous gonadotropin stimulation of Leydig cells, together with the negative paracrine action of macrophages on these testosterone producing cells, could result in the damage of steroidogenesis and deficit of testosterone* in situ*.

## Figures and Tables

**Table 1:** Testis volume, score of spermatogenesis, and hormone values in the control group (OA) and in patients with nonobstructive azoospermia (NOA).

	OA Vo (cm^3^)	NOA Vo (cm^3^)	OA score	NOA score	OA FSH (UI/L)	NOA FSH (UI/L)	OA LH (UI/L)	NOA LH (UI/L)	OA T (nmol/L)	NOA T (nmol/L)
*ẍ*	19.737	8.123	9.582	4.131	8.55	19.74	5.04	10.06	11.46	12.49
s	1.866	3.707	0.163	2.052	1.628	1.58	3.10	4.12	3.838	5.89
SE	0.311	0.618	0.027	0.342	3.51	2.59	0.93	0.92	1.16	1.32
Max	22.43	16.34	8.857	8.171	10.60	51.9	10.00	18	18.60	27.1
Min	16.34	4.03	8.314	1	2.23	5	1.60	4.97	5.20	2.6
*P* (OA/NOA)	<0.001		<0.001		<0.01		<0.001		>0.05	

*ẍ*: mean; s: standard deviation; SE: standard error of mean; Max: maximal value; Min: minimal value; *P* (OA/NOA): statistical significance between OA and NOA.
